# The Effect of the AQP1 Gene Knockout on the Diversity, Composition and Function of Gut Microbiota in Mice with Heart Failure

**DOI:** 10.3390/biology14070815

**Published:** 2025-07-04

**Authors:** Haotian Li, Yubo Li, Jianqin Yang, Yanjun Liu

**Affiliations:** 1 School of Traditional Chinese Medicine, Beijing University of Chinese Medicine, Beijing 100029, China; 20240931064@bucm.edu.cn; 2 Institute of Basic Theory of Traditional Chinese Medicine, China Academy of Chinese Medical Sciences, Beijing 100700, China; liyb@ibtcm.ac.cn; 3 Department of Digestive Disorders, Beijing Key Laboratory of Diagnosis and Treatment of Functional Gastrointestinal Diseases of Traditional Chinese Medicine, Beijing 100102, China

**Keywords:** AQP1, gene knockout, gut microbiota, heart failure

## Abstract

Introduction: This study aims to elucidate the impact of AQP1 on cardiac function and the intestinal microbiota in mice with chronic heart failure and to further investigate the broad effects of AQP1 on the gut microbiota composition in these mice. Methods: AQP1 knockout mice were used as the experimental group, with wild-type mice serving as the control group. The study evaluated the effects of AQP1 on various physiological parameters, including blood pressure, heart rate, cardiac function, cardiac color Doppler ultrasound, and 24 h urine collection. Additionally, the high-throughput sequencing of gut microbiota was performed to identify key microbial communities. Results: The deletion of the AQP1 gene did not significantly alter key cardiovascular metrics such as systolic blood pressure (SBP), mean blood pressure (MBP), or left ventricular mass (LV mass). However, we found that AQP1 knockout affected 24 h urine output in mice. Echocardiography results showed that AQP1 expression influenced LV mass, LVAW; d, and LVPW; s. Moreover, substantial differences were observed in the intestinal microbiota profiles between AQP1 knockout mice with heart failure and their wild-type counterparts. These findings suggest that AQP1 may contribute to cardiac dysfunction in mice with chronic heart failure through the regulation of gut microbiota. Conclusion: Our investigation provides initial insights into the role of AQP1 in modulating the intestinal microbiota in a murine model of heart failure. However, the precise mechanisms underlying this association require further exploration and detailed analysis.

## Simple Summary

Aquaporin 1 (AQP1) is widely expressed in myocardial cells and is closely associated with the physiology and pathology of heart failure progression. Both aquaporins (AQPs) and gut microbiota are now recognized as key factors in the pathogenesis of heart failure, which is closely linked to alterations in the richness and diversity of the gut microbiota. Changes in gut microbiota composition and the impairment of gut barrier function lead to chronic low-grade inflammation, which further compromises heart function. Our study explores the effects of AQP1 deficiency on physiological parameters such as the baseline rate of heart failure and blood pressure and aims to clarify the relationship between AQP1 deficiency and alterations in the gut microbiota.

## 1. Introduction

Heart failure (HF) represents an advanced stage of various cardiovascular diseases and poses a significant global health challenge due to its high prevalence and poor prognosis.

Aquaporins are a family of membrane proteins embedded in the cell membrane that facilitate the passage of water molecules [1,2], playing a critical role in water transport and metabolism within the human body. To date, 13 types of aquaporins have been identified, among which Aquaporin 1 (AQP1) is essential for water transport across the cell membrane. AQP1 is widely expressed in myocardial cells and is closely associated with the physiology and pathology of HF progression.

Previous studies have demonstrated that alterations in AQP1 correlate with intracellular water overload, myocardial cell swelling, and impaired cardiac function. As HF symptoms worsen, AQP1 expression in myocardial cells decreases accordingly [3,4]. Currently, the RNA sequencing of human cardiomyocytes has identified AQP1 as the predominant aquaporin subtype, highlighting its potential as a therapeutic target for HF treatment. Moreover, both aquaporins (AQPs) and the gut microbiota are increasingly recognized as key contributors to the pathogenesis of HF [5]. This relationship is tightly linked to shifts in the richness and diversity of the gut microbiota. Circulatory dysfunction often coincides with changes in microbial species and the disruption of gut barrier integrity, resulting in chronic low-grade inflammation that further impairs cardiac function and circulation.

To investigate the systemic effects of AQP1 deficiency on gut microbiota composition and function in heart failure, this study employed 16S rRNA sequencing to compare the gut microbiota of AQP1 knockout (AQP1^−/−^) mice with their wild-type (AQP1^+/+^) counterparts. We analyzed alpha and beta diversity, and conducted functional prediction to assess the impact of AQP1 deficiency on gut microbial classification and function. Complementary assessments, including cardiac ultrasound evaluation, blood pressure measurement, and the analysis of 24 h urine volume, were also conducted to gain a comprehensive understanding of the systemic effects of AQP1 deficiency in the context of heart failure.

## 2. Materials and Methods

### 2.1. Construction of AQP1 Knockout Mice

The AQP1 transcript targeted was AQP1-201 (Ensemble: ENSMUST00000004774). Four gRNAs were designed to target exons 1–4 of AQP1 (gRNA1: GAGGCAACCGTTGCTCTAATAGG; gRNA2: GCCCACGAGCACACGTGGATGGG; gRNA3: TGTCTGCATCCATCGCAGCGAGG; gRNA4: CCGTGTAACATGGCCCCGACTGG). Cas proteins and gRNAs were microinjected into the fertilized eggs of C57BL/6N mice, resulting in three F0 generation mice. Positive knockouts were identified by PCR amplification and sequencing using primers flanking the knockout region: forward primer F1 (5’-TCAGCCTGAGAACTTCAGATTACTA-3’) and reverse primer R1 (5’-ATGATGAACTAAGGGCATCCAAAC-3’). The expected knockout band size was 599 bp.

F0-positive mice were crossed with wild-type C57BL/6N mice, producing three F1 heterozygous AQP1^+/−^ mice. F1 heterozygotes were intercrossed to generate F2 homozygous knockout mice (AQP1^−/−^). F2 homozygous mice were subjected to IVF experiments, yielding 44 males and 36 females homozygous for AQP1 knockout (AQP1^−/−^). For genotyping heterozygotes, the primers used included F1 (5’-TCAGCCTGAGAACTTCAGATTACTA-3’) and R2 (5’-CTTCTTGATTTCACTGGCCATGCT-3’) located within the knockout region. Homozygous knockouts showed no band, whereas heterozygotes yielded a 580 bp band.

### 2.2. RT-PCR

Heart tissue samples were collected, and total RNA was extracted using a spin-column RNA extraction kit. cDNA was synthesized via reverse transcription. Real-time PCR was performed using SYBR Premix Ex Taq(G3320 Servicebio, Wuhan, China), with β-actin as the internal reference gene. Target gene expression levels were quantified using the ΔΔCt method. The primer sequences used were forward (GCCTTGTCTGGGGCAGTAAT) and reverse (GACAACTAGCAGGTGGGTCC). Independent experimental batches were repeated twice to ensure reproducibility.

### 2.3. Western Blotting

Total protein was extracted from the myocardial tissue of each mouse and quantified. The specific steps are as follows: after taking 100 mg of myocardial tissue, cell lysates were prepared by mixing centrifuged cells with an RIPA buffer and 5 × protein loading buffer and then stored at −80 °C after sonication. Proteins were separated by electrophoresis and transferred to membranes, which were blocked with 5% skimmed milk for 1.5 h. Membranes were washed three times with TBST (10 min each), then incubated overnight at 4 °C with the primary antibody AQP1 (1:10,000, GB11310-1, Servicebio, Wuhan, China). After washing, the membranes were incubated with a secondary antibody for 1 h, followed by three TBST washes (10 min each). Protein bands were analyzed for intensity using ImageJ software Version 1.54p (Media Cybernetics, Bethesda, MD, USA) for densitometry quantification. Independent experimental batches were repeated twice to ensure reproducibility.

### 2.4. Establishment and Grouping of Mouse Heart Failure Models

Based on the literature reports and preliminary experiments, chronic HF models were established via the intraperitoneal injection of isoproterenol (ISO) at 7.5 mg/kg [6,7]. Both AQP1^−/−^ and AQP1^+/+^ mice, with equal numbers of males and females, were subjected to the procedure. One month after modeling, echocardiography was performed to confirm the successful induction of HF. Mice with confirmed HF were then divided into four groups: AQP1^−/−^ control and model groups and AQP1^+/+^ control and model groups, with 10 mice per group (half male, half female).

### 2.5. Detection of Cardiac Function Changes in Mice with HF

Mice were anesthetized with isoflurane (ISO) using an air anesthesia machine and secured in the supine position on a mouse platform. The precordial skin was prepared, and a cardiac ultrasound was performed using a Vivid 7 Dimension color Doppler ultrasound system with a 13 MHz probe frequency, a depth of 3.5 cm, and a speed of 200 mm/s. A suitable short-axis two-dimensional image of the parasternal left ventricle was obtained. M-mode sampling lines were positioned perpendicular to the ventricular septum and the posterior wall of the left ventricle at the level of the papillary muscles. Measurements included fractional shortening (FS), and ejection fraction (EF), etc. The average values from three consecutive cardiac cycles were recorded. This experiment was repeated across three independent batches to confirm reproducibility.

### 2.6. Measure Blood Pressure

Blood pressure was measured non-invasively using a mouse tail-cuff blood pressure monitor (Kent Scientific, Torrington, CT, USA). Mice were restrained and placed on a heated platform for temperature maintenance. The mouse tail was connected to the dedicated measuring device, and the blood pressure measurement software was initiated. A total of 20 measurements were taken for each mouse, including 5 pre-cycles, recording the heart rate (HR), mean blood pressure (MBP), systolic blood pressure (SBP), and diastolic blood pressure (DBP) for each group. The experiment was conducted across three independent batches to ensure reproducibility.

### 2.7. 24-Hour Urine Volume Test

After successful modeling, the mice were transferred to metabolic cages, fasted without water, and 24 h urine output was collected from each group of mice.

### 2.8. Sequencing of Mouse 16S rDNA Gut Microbiota

#### 2.8.1. Genomic DNA Extraction and Quality Inspection

Total DNA was checked using a Thermo NanoDrop 2000 UV microvolume spectrophotometer and 1% agarose gel electrophoresis.

#### 2.8.2. Primer Design and Synthesis

The 16S rDNA amplification region was selected for the V3-V4 region, and the universal primers used were F341 and R806. The design of specific primers was completed by adding the index and adapter sequences suitable for HiSeq2500 PE250 sequencing at the 5’ end of the universal primer.

Forward primer (5’–3’): ACTCCTACGGGRSGCAGCAG (F341).

Eeverse primer (5’–3’): GGACTACVVGGGTATCTAATC (R806).

#### 2.8.3. PCR Amplification and Product Purification

PCR amplification was performed using the KAPA HiFi HotStart ReadyMix PCR kit with a high-fidelity enzyme, using diluted genomic DNA as the template to ensure precise and efficient amplification. PCR products were verified by 2% agarose gel electrophoresis. Target bands were excised and purified using the AxyPrep DNA Gel Recovery Kit (Axygen Corporation, Union City, CA, USA). After purification, library quality was assessed using a Thermo NanoDrop 2000 UV microvolume spectrophotometer and 2% agarose gel electrophoresis.

Following the quantification and normalization of PCR products, libraries passing quality control were quantified using Qubit and pooled according to the required sequencing depth for each sample.

#### 2.8.4. Illumina Sequencing

Sequencing was performed using the Illumina HiSeq PE250 platform according to standard protocols.

### 2.9. Statistical Analysis

Statistical analyses were conducted using SPSS version 21.0. Continuous data are presented as the mean ± standard deviation (x ± s). One-way ANOVA was used for group comparisons. For homogeneity of variance, the Least Significant Difference (LSD) test was applied; for heterogeneity of variance, Dunnett’s T3 test was used. A *p*-value of less than 0.05 was considered statistically significant.

## 3. Results

### 3.1. Western Blotting and PCR

**Figure 1:**
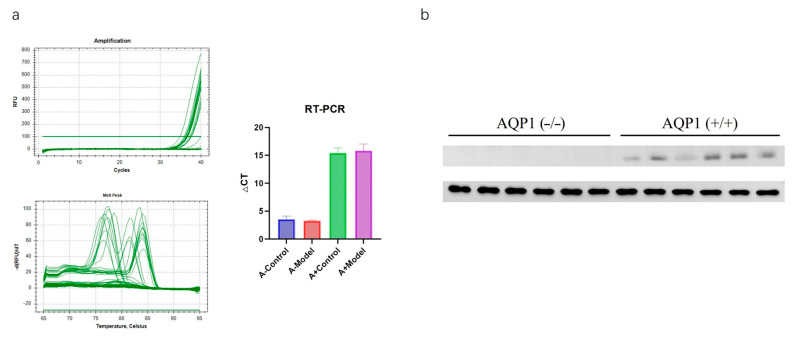
(**a**) RT-PCR result; (**b**) WB result.

We conducted PCR and WB testing to further confirm the knockout of the gene. (Figure 1).

### 3.2. Blood Pressure and Heart Rate

**Figure 2:**
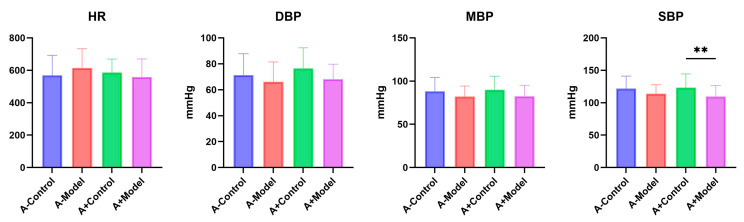
The blood pressure and heart rate statistics of AQP1^−/−^ mice versus AQP1^+/+^ mice. A-Control is a control group of AQP1 knockout (AQP1^−/−^) mice; A-Model is an HF group of AQP1 knockout (AQP1^−/−^) mice; A+Control is a control group of wild-type (AQP1^+/+^) mice; and A+Model is an HF group of wild-type (AQP1^+/+^) mice (**: <0.01).

Following the successful modeling of AQP1^−/−^ mice and AQP1^+/+^ mice (Figure 2), we compared HR, DBP, and MBP between the control and model groups of these two groups of mice. No significant differences were observed in these parameters across the groups. However, for SBP, no significant difference was found between the control and model groups in AQP1^−/−^ mice. In contrast, a significant difference was observed between the corresponding groups in AQP1^+/+^ mice (*p* < 0.05). Detailed information could be found in Supplementary Materials.

These results suggest that AQP1 knockout does not significantly affect heart rate or blood pressure in mice with heart failure.

### 3.3. 24-Hour Urine Output

**Figure 3:**
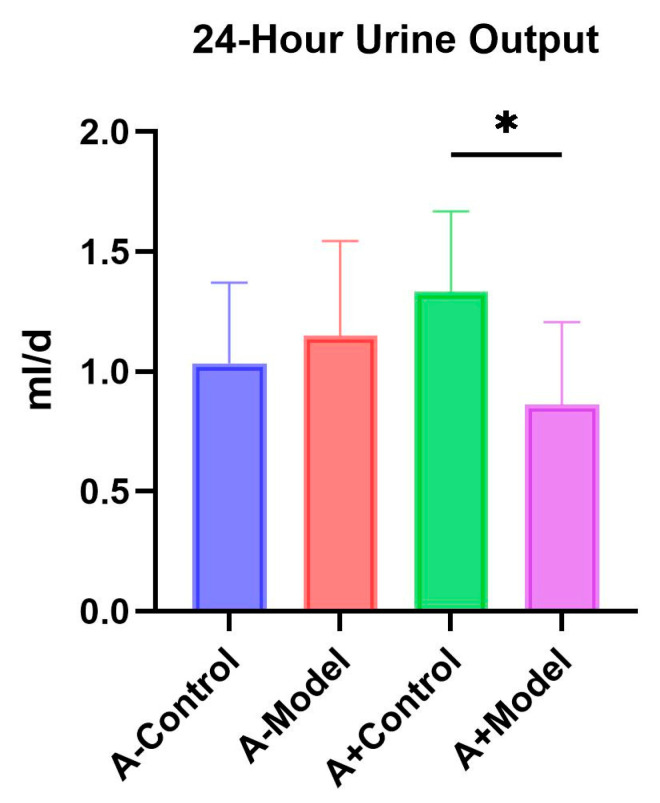
The 24 h urine output. AQP1^−/−^ mice versus AQP1^+/+^ mice. A-Control is a control group of AQP1 knockout (AQP1^−/−^) mice; A-Model is an HF group of AQP1 knockout (AQP1^−/−^) mice; A+Control is a control group of wild-type (AQP1^+/+^) mice; and A+Model is an HF group of wild-type (AQP1^+/+^) mice (*: <0.05).

There was no significant difference in 24 h urine output between the control and model groups of AQP1^−/−^ mice (*p* > 0.05) (Figure 3), whereas a significant difference was observed between the control and model groups of AQP1^+/+^ mice (*p* < 0.05). These results suggest that AQP1 knockout may increase after 24 h urine output in mice; however, this trend did not reach statistical significance. Detailed information could be found in Supplementary Materials.

### 3.4. Echocardiographic Results

**Figure 4:**
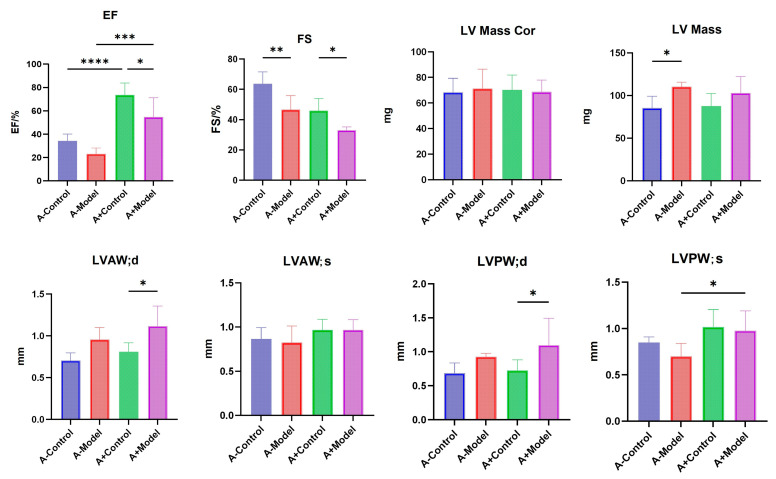
A statistical diagram of the cardiac ultrasounds of AQP1^−/−^ mice versus AQP1^+/+^ mice. A-Control is a control group of AQP1 knockout (AQP1^−/−^) mice; A-Model is an HF group of AQP1 knockout (AQP1^−/−^) mice; A+Control is a control group of wild-type (AQP1^+/+^) mice; and A+Model is an HF group of wild-type (AQP1^+/+^) mice (*: < 0.05; **: <0.01; ***: <0.001; ****: <0.0001).

The left ventricular ejection fraction was significantly different between the control and model groups in both AQP1^−/−^ and AQP1^+/+^ mice (*p* < 0.05). Similarly, there was a significant difference in left ventricular fractional shortening (FS %) between the control and model groups in both AQP1^−/−^ and AQP1^+/+^ mice (*p* < 0.05). These findings indicate that the intraperitoneal injection of ISO effectively establishes an HF model in mice. Additionally, we observed a significant difference in the left ventricular (LV) mass between the control and model groups of AQP1^−/−^ mice. A significant difference in left ventricular anterior wall thickness at the end-diastole (LVAW; d) was found between the control and model groups of AQP1^+/+^ mice (*p* < 0.05), as well as between the AQP1^−/−^ and AQP1^+/+^ model groups (*p* < 0.05). Moreover, there was a significant difference in left ventricular posterior wall thickness at the end-systole (LVPW; s) between the control and model groups of AQP1^+/+^ mice (*p* < 0.05). In summary, these results suggest that AQP1 expression affects LV mass, LVAW; d, and LVPW; s (Figure 4). Detailed information could be found in Supplementary Materials.

### 3.5. Microbiota Sequencing Results Based on 16SDNA of Four Groups of Mice

**Figure 5:**
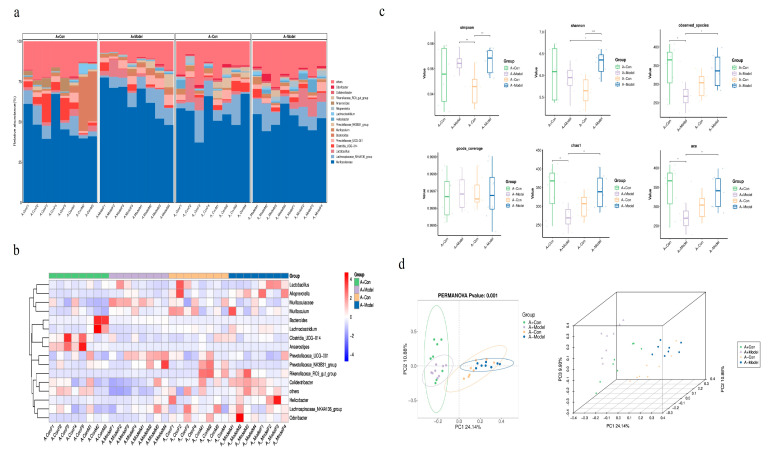
The gut microbiota composition in AQP1^−/−^ mice versus AQP1^+/+^ mice. A-Con is a control group of AQP1 knockout (AQP1^−/−^) mice; A-Model is an HF group of AQP1 knockout (AQP1^−/−^) mice; A+Con is a control group of wild-type (AQP1^+/+^) mice; and A+Model is an HF group of wild-type (AQP1^+/+^) mice. (**a**) A relative abundance histogram showing species composition at the genus level in the HF and control groups of both AQP1 knockout and wild-type mice; (**b**) a relative abundance bar chart comparing species composition between HF and control groups in both AQP1 knockout and wild-type mice; (**c**) the alpha diversity analysis of gut microbiota across HF and control groups in AQP1 knockout and wild-type mice; and (**d**) the beta diversity analysis (PCA) of gut microbiota composition among HF and control groups in AQP1 knockout and wild-type mice (*: <0.05; **: <0.01; ***: <0.001).

Using high-resolution ASV (amplicon sequence variant) data analysis (Figure 5a), histograms were generated to compare gut microbial diversity in AQP1^−/−^ and AQP1^+/+^ mice across both control and model groups.

The analysis revealed that in control groups, the number of ASVs was significantly higher in wild-type mice (2619) compared to knockout mice (2259). In contrast, within the model groups, AQP1^−/−^ mice exhibited a higher ASV count (2568) than their wild-type counterparts (2055). These findings indicate that AQP1 knockout reduces microbial richness under normal conditions but appears to preserve or even increase it under HF conditions.

Alpha diversity analysis (Figure 5c) showed no significant differences in Good’s coverage index among the groups, suggesting that sequencing depth was consistent. However, the Shannon index showed significant differences between the control groups of AQP1^−/−^ and AQP1^+/+^ mice (*p* < 0.05), and also between the control and model groups within the AQP1^−/−^ genotype. Similarly, the Simpson index revealed significant differences between the control and model groups in AQP1^−/−^ mice. Both the Chao1 and ACE indices indicated significant changes in species richness between the control and model groups in both AQP1^−/−^ and AQP1^+/+^ mice. Collectively, these results demonstrate that AQP1 deficiency leads to marked alterations in gut microbial richness and diversity in the context of heart failure.

Beta diversity analysis using principal component analysis (PCA) (Figure 5d) revealed distinct shifts in gut microbiota composition driven by both AQP1 gene status and the HF model. Notably, the control groups of AQP1^−/−^ and AQP1^+/+^ mice clustered separately, indicating clear compositional differences. The model groups showed limited overlap with their respective controls but did not overlap with each other, highlighting substantial divergence in microbial communities. These patterns suggest that both AQP1 deficiency and the HF condition distinctly shape the gut microbiota, with the disease model further constraining microbial diversity.

## 4. Discussion

In this study, we examined blood pressure, 24 h urine output, cardiac function, and gut microbiota composition in both AQP1^−/−^ and AQP1^+/+^ mice under normal and HF conditions. Our results showed no significant differences in HR, DBP, or MBP between the control and HF groups in either AQP1^−/−^ or AQP1^+/+^ mice. Hypertension is a known risk factor for HF, as it increases cardiac afterload, promotes cardiomyocyte hypertrophy and interstitial fibrosis, and leads to ventricular hypertrophy and dilation, thereby contributing to the development and progression of HF [8]. Based on the findings from AQP1^−/−^ mice, it appears that AQP1 deletion does not significantly affect basic cardiovascular parameters such as HR and BP.

Regarding renal function, we observed a significant difference in 24 h urine output between the control and HF groups in AQP1^+/+^ mice, whereas no significant difference was detected in AQP1^−/−^ mice across the same conditions. Additionally, there was no significant difference in 24 h urine output between the AQP1^−/−^ and AQP1^+/+^ mice within the control group. Although not statistically significant, AQP1 knockout appeared to influence urinary output. Previous studies have reported a positive correlation between 24 h urine volume and HF severity [9]. These findings suggest that the AQP1 expression is closely associated with changes in urine output and that the modulation of AQP1 levels may offer a potential therapeutic strategy to improve fluid regulation in HF.

Cardiac ultrasound analysis revealed a significant reduction in EF% between the control and model groups for both AQP1^−/−^ and AQP1^+/+^ mice, indicating impaired left ventricular systolic function under HF conditions. Similarly, FS % was significantly decreased in the HF groups compared to their respective controls in both genotypes. These results suggest that AQP1 knockout may influence left ventricular function, particularly in terms of contractility and wall motion. Moreover, significant differences were observed in LVAW; d between the control and model groups of AQP1^+/+^ mice. Additionally, the left ventricular mass was significantly altered in the HF group of AQP1^−/−^ mice. A significant difference in LVAW; d was also noted between AQP1^−/−^ and AQP1^+/+^ mice under HF conditions (*p* < 0.05). Furthermore, LVPW; s differed significantly between the control and HF groups in AQP1^+/+^ mice. Collectively, these echocardiographic findings indicate that AQP1 expression plays a role in regulating the structural and functional cardiac changes associated with HF.

These results indicate that AQP1 expression has a measurable impact on the thickness of the anterior LVAW; d, as well as on the posterior wall of the left ventricle during both systole and diastole phases. Additionally, a significant difference in LV mass was observed between the control and HF groups in AQP1^−/−^ mice, suggesting that AQP1 expression influences both the systolic and diastolic function of the left ventricle. This, in turn, may exacerbate myocardial injury and worsen HF symptoms. Previous studies have demonstrated that AQP1 plays a key role in the regulation of myocardial edema. The overexpression of AQP1 can increase the severity of myocardial edema, thereby impairing cardiac function. Research has shown that myocardial ischemia and infarction can activate AQP1, disrupting the cytoskeletal integrity of cardiomyocytes and increasing the fragility of the myocardial membrane. This results in enhanced membrane permeability, allowing water molecules to enter the cell through the AQP1 channel, leading to cardiomyocyte swelling [10]. In contrast, the downregulation or inhibition of AQP1 expression has been shown to attenuate myocardial edema and fibrosis, potentially delaying the progression of HF [11]. In the present study, HF was induced in both AQP1^−/−^ and AQP1^+/+^ mice, and cardiac ultrasound findings confirmed that the influence of AQP1 on cardiac function aligns with previously reported data. These results further support the hypothesis that AQP1 is involved in regulating cardiac structural and functional changes during HF.

Furthermore, gut microbiota analysis revealed that AQP1 gene knockout significantly influences microbial diversity and richness. The number of ASVs in the control group of AQP1^+/+^ mice was significantly higher than the number in AQP1^−/−^ mice. Interestingly, in the HF model group, the ASV count in AQP1^−/−^ mice exceeded that of their respective controls. Alpha diversity metrics—including the Shannon index, Simpson index, Chao1, and ACE index—showed significant differences between AQP1^−/−^ and AQP1^+/+^ mice, indicating that AQP1 expression modulates the composition of gut microbiota under physiological conditions and has a substantial impact on microbial diversity and richness in HF. These findings suggest that AQP1 gene deletion may influence cardiac function partly by altering the gut microbial ecosystem. Beta diversity analysis (PCA) further demonstrated distinct clustering patterns. In the control group, microbial communities of AQP1^−/−^ and AQP1^+/+^ mice showed no overlap, indicating substantial differences. In the HF model groups, both AQP1^−/−^ and AQP1^+/+^ mice formed clusters that were distinct from their respective controls but did not overlap with each other, suggesting significant shifts in microbial composition induced by both HF and AQP1 status. Collectively, these findings indicate that AQP1 expression significantly alters the gut microbiota profile in both healthy and HF conditions.

## 5. Conclusions

Systemic AQP1^−/−^ and AQP1^+/+^ mice were used to model HF, revealing certain differences in blood pressure, 24 h urine output, cardiac function, and gut microbiota. The loss of the AQP1 protein resulted in an abnormal decrease in blood pressure and an increase in 24 h urine output. Notably, abnormalities in the thickness of the anterior wall of the left ventricle during the diastole phase, as well as in the systolic and diastolic thickness of the left ventricular posterior wall and left ventricular mass index, contributed to aggravated myocardial dysfunction, thereby influencing HF symptoms. Based on the analysis of intestinal microbiota, this study demonstrated that AQP1 gene knockout significantly affected the gut microbial composition in HF mice. Moreover, it confirmed that the effects of gene knockout and HF modeling on the gut microbiota are both independent and additive. This study also assessed the overall composition of microbiota; however, further investigation into specific microbial metabolites, such as bile acids and short-chain fatty acids, is needed to clarify whether these substances mediate the impact of AQP1 on cardiac function. Overall, these findings offer new insights into the development of chronic HF treatment strategies targeting gut microbiota.

## Data Availability

The original contributions presented in this study are included in the article/Supplementary Materials. Further inquiries can be directed to the corresponding author.

## Supplementary Materials

The following supporting information can be downloaded at: https://www.mdpi.com/article/10.3390/biology14070815/s1.
