# Supplementary figures and images for: The Effect of the AQP1 Gene Knockout on the Diversity, Composition and Function of Gut Microbiota in Mice with Heart Failure
Source: Biology (Basel). 2025 Jul 4;14(7):815. doi: 10.3390/biology14070815 (PMC12292741; doi:10.3390/biology14070815)

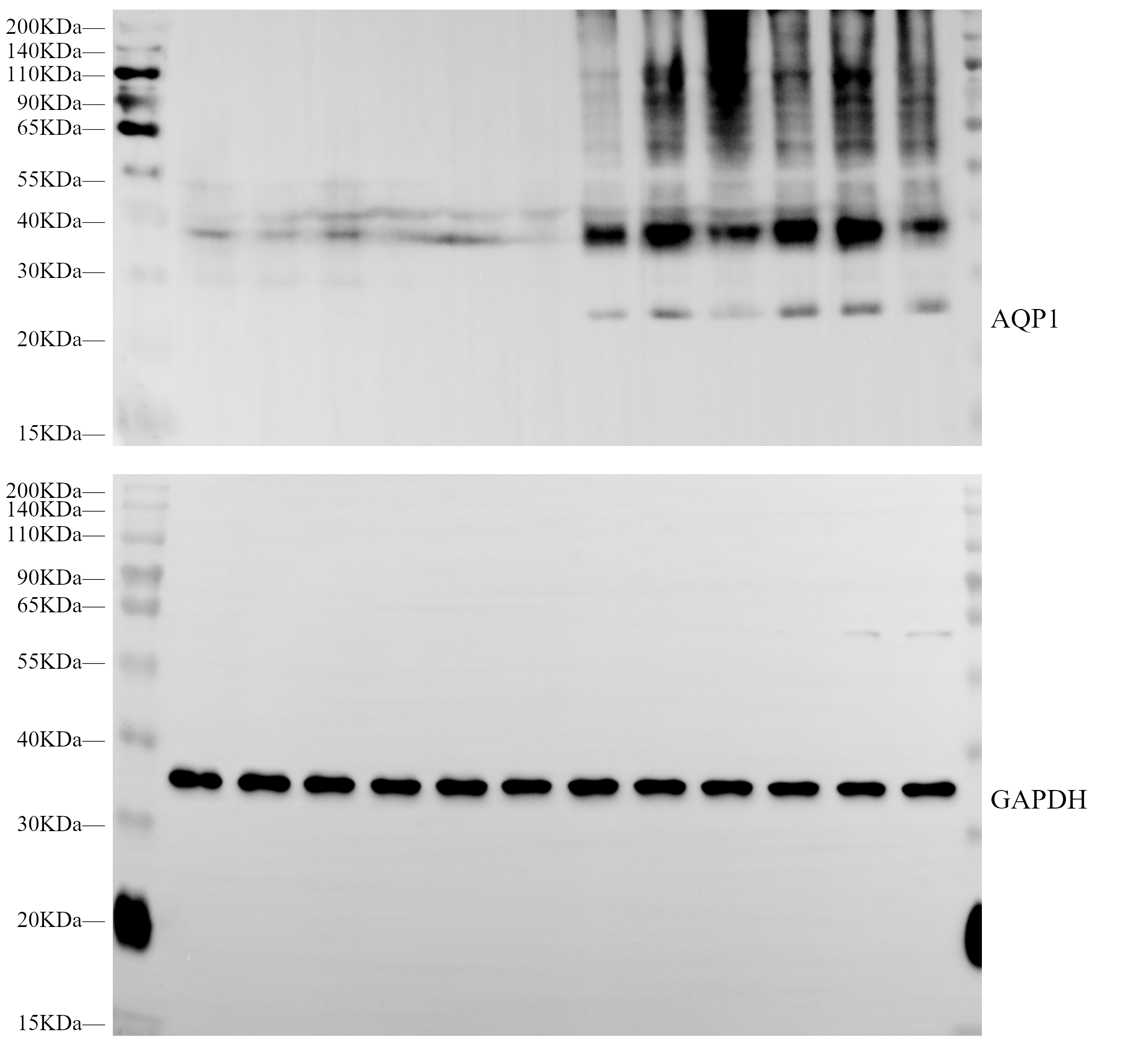

Supplement: Supplementary file 1 [file biology-14-00815-s001.zip › WB.tif]
